# Experiences of recent weight-management attempts: insights from a web-based cross-sectional study

**DOI:** 10.3389/fpubh.2025.1709949

**Published:** 2025-12-17

**Authors:** Jamil Ahmed, Aseel AlSaleh, Amer Almarabheh, Layan Abdulmunem AlAbbas, Eman Mohammed Alyaseen, Aysha Almuqahwi, Mohamed Alqahtani

**Affiliations:** College of Medicine and Health Sciences, Arabian Gulf University, Manama, Bahrain

**Keywords:** obesity, barriers to weight loss, sustaining weight loss, physical activity, exercise, diet, Bahrain

## Abstract

**Background:**

Understanding perceived barriers and facilitators in this context is essential for designing effective interventions. This study aimed to determine perceived barriers to weight loss and maintaining an optimum weight among Bahraini adults who have previously tried to lose weight.

**Methods:**

A cross-sectional study was conducted between March and July 2023 using an online questionnaire targeting Bahraini adults who had attempted weight loss within the past year. Individuals with a history of surgical interventions for obesity were excluded. The questionnaire was pretested for consistency and administered in both English and Arabic. Participants were recruited via social media platforms, yielding 1,453 complete responses.

**Results:**

The prevalence of overweight and obesity among participants was 78.2%, with a higher crude prevalence observed among women (81.4%) compared to men (66.7%). However, after adjusting for sociodemographic and behavioral factors, female participants had lower odds of obesity (AOR = 0.50, 95% CI: 0.37–0.67), indicating that the higher unadjusted prevalence among women was influenced by confounding factors such as age and occupation distribution. Increasing age was strongly associated with higher odds of obesity, particularly among individuals aged 36–45 years (AOR = 3.37, 95% CI: 1.91–5.95) and >45 years (AOR = 3.59, 95% CI: 1.75–7.37), compared with those aged <25 years. Marital status and employment also showed significant associations: married individuals (AOR = 1.79, 95% CI: 1.30–2.46), unemployed participants (AOR = 2.36, 95% CI: 1.48–3.76), and retired participants (AOR = 7.67, 95% CI: 2.86–20.53) had higher odds of obesity. Weight-loss behaviors were also associated with obesity status: those who dieted (AOR = 2.53, 95% CI: 1.81–3.54), exercised (AOR = 1.48, 95% CI: 1.07–2.04), or used medications (AOR = 5.23, 95% CI: 2.93–9.35) to lose weight had higher odds of obesity. Participants who held neutral views regarding lack of exercise as a cause of weight gain demonstrated lower odds of obesity (AOR = 0.59, 95% CI: 0.39–0.90).

**Conclusion:**

This study identifies that perceptions of obesity are driven by sociodemographic and lifestyle factors, with women reporting greater challenges and a stronger reliance on conventional weight-loss methods. Physical inactivity and unhealthy dietary practices emerged as key barriers to effective weight management.

## Introduction

Countries of the Gulf Cooperation Council (GCC), Saudi Arabia, Bahrain, Kuwait, Oman, Qatar, and the United Arab Emirates, have some of the highest burdens of obesity and physical inactivity rates. In Bahrain, 70.3% of the population is overweight, and 32.6% have obesity (1). Approximately 67.8% perform no physical activity (2). A national public survey revealed high prevalence rates of hypertension (42.7%), hypercholesteremia (40.2%), and type 2 diabetes (14.3%) among Bahrainis. Recent studies found that diabetes prevalence has increased by 21.84% (3, 4). In Bahrain, non-communicable diseases (NCDs) are the top causes of morbidity and mortality (1, 4), accounting for more than one million visits to the health centers annually (4, 5). Currently, approximately 200,000 Bahrainis are affected by NCDs, with cardiovascular diseases affecting over 122,000 individuals and diabetes affecting over 60,000 (5). This high prevalence of obesity and NCDs in the country has even prompted the establishment of nutritional clinics aimed at tackling obesity and encouraging a healthier lifestyle (6, 7). Nevertheless, the population-based obesity rates remain persistently high.

Increased exposure to an obesogenic environment significantly impacts dietary behaviors and is a major driving force behind rising obesity levels. Individuals interact within micro-environments (e.g., home, fast food restaurants, and school) that are closely linked to macro-environments (e.g., the food industry, societal attitudes and beliefs, and government) (8). Consumption of energy-rich fast foods is higher during special occasions in the region. For example, during Ramadan and other religious and cultural events, it is common to indulge in high-energy meals. Although fasting can increase insulin sensitivity and promote weight loss, one study reported that approximately two-thirds of participants (59.5%) gained weight during the holy month, largely due to excess consumption of carbohydrates, fats, and sugary food (9). Another study found that 37% of Saudi participants reported increased food expenditures, mainly due to celebrations and social gatherings.

Overweight and obesity have emerged as major public health challenges worldwide, contributing substantially to the burden of NCDs and escalating health system costs. In the Gulf region, including Bahrain, rapid urbanization, dietary transitions, and sedentary lifestyles have accelerated the rise in obesity prevalence. To develop effective strategies for obesity prevention and management, it is essential to understand the factors associated with weight loss and maintenance. Exploring perceptions of the drivers of weight gain, motivations to lose weight, and efforts to sustain in populations with cultural and traditional contexts distinct from those of Western societies may help identify key areas for policy development and open new avenues for research and intervention. This study focuses on Bahraini adults who have attempted weight loss within the past year, aiming to explore perceived challenges to achieving and sustaining an optimum weight, and to identify locally relevant evidence to inform public health strategies for obesity prevention.

## Methods

### Study design

This cross-sectional study was conducted using an online questionnaire. The participants who had attempted weight loss within the past year were invited to complete the survey.

### Study population and sample size

All adults who had attempted weight loss within the previous year were eligible to participate in the study. Individuals who had undergone surgical interventions for obesity were excluded. Questionnaire responses collected between March and July 2023 were considered eligible for inclusion in the analysis.

### Ethical considerations

The study was approved by the Research and Ethics Committee of the College of Medicine and Health Sciences, Arabian Gulf University (Approval No. E38-PI-6-22). Data collection was conducted in accordance with the principles of anonymity and informed consent. The initial page of the online questionnaire provided participants with standardized information regarding the study objectives, their rights as participants, and the option to withdraw at any stage without justification. Participants provided consent by selecting the “agree” option before proceeding with the survey. All responses were treated confidentially, and appropriate data security measures were maintained throughout the study.

### Data collection

The questionnaire was developed following a comprehensive review of the literature, ensuring inclusion of variables relevant to the study objectives. Content validity was quantitatively assessed using the content validity index (CVI) approach. Ten experts in public health, obesity research, and behavioral science independently rated the relevance and clarity of each item on a 4-point scale (1 = not relevant to 4 = highly relevant). The item-level CVI values ranged from 0.83 to 1.00, while the scale-level CVI was 0.92, indicating excellent content validity. To evaluate internal consistency, the questionnaire was pretested on 15 participants from the target population. The overall Cronbach’s *α* was 0.87, reflecting a high level of internal consistency. Based on expert feedback and pilot testing, minor wording revisions were made to enhance clarity and cultural appropriateness prior to the final administration.

English and Arabic translated versions of the questionnaire were administered. The original English questionnaire was translated into Arabic by a bilingual expert, and discrepancies were reviewed and resolved by a panel of bilingual researchers to ensure semantic and conceptual equivalence. A total of 197 participants (13.6%) completed the English version, while 1,256 participants (86.4%) responded to the Arabic version. Height and weight were self-reported, and body mass index (BMI) was calculated as weight in kilograms divided by height in meters squared. Overweight and obesity were defined using the World Health Organization (WHO) cutoffs. Participants were invited by the research team through widely used social media platforms (Instagram, WhatsApp, and Twitter) to complete the structured questionnaires. Data collection was conducted between March and July 2023, during which reminders were sent through social media to the participants. Recruitment was conducted by posting the survey link on 10 pages and sharing it across 30 online groups, which may have skewed the sample toward younger and more digitally active participants.

### Data analysis

Descriptive statistics and chi-squared tests were used to examine associations between categorical variables, with obesity status (BMI > 30) as the outcome (*p* < 0.05). Behavioral variables were operationally defined as follows: (a) “Dieting” was recorded as a binary variable (yes/no), based on participants’ self-report of any intentional efforts to restrict food intake or follow a specific diet plan in the past month. (b) “Exercise” was similarly coded as a binary variable, indicating whether the participant engaged in any form of physical activity, such as walking, sports, or gym workouts, during the same period. (c) “Medication use” referred to the self-reported use of any prescribed or over-the-counter medications related to weight control, metabolic health, or chronic disease management, and was treated as a binary variable. Missing data were minimal (<5%) and were handled using listwise deletion after confirming that the data were missing completely at random via Little’s test. Univariate logistic regression analyses were performed to identify candidate variables associated with obesity, and variables with *p* < 0.20 were considered for inclusion in the multivariable logistic regression model. The adjusted model included covariates based on theoretical relevance and univariate significance, including sex, age group, marital status, education level, occupation, and monthly income, as well as behavioral factors (dieting, exercise, medication use, and surgery) and perceived behavioral factors (“eating unhealthy food” and “lack of exercise”). Multicollinearity was examined using variance inflation factors (VIFs), with all values <2.0, indicating no multicollinearity issues. Model fit was evaluated using the Hosmer–Lemeshow test (*χ*^2^ = 7.82, *p* = 0.45), Nagelkerke *R*^2^ (0.312), and likelihood ratio test (*χ*^2^ = 226.5, *p* < 0.001), confirming satisfactory fit. Adjusted odds ratios (AORs) with corresponding 95% confidence intervals (CIs) were reported for all predictors. Data were analyzed using SPSS version 30 and R software version 4.3.2.

## Results

The study included 1,453 participants, comprising 62.8% women and 37.2% men. Most participants were aged ≤25 years (35.9%), 25–35 years (31.6%), 36–45 years (18.3%), and >45 years (14.2%), with a mean age of 32.55 ± 12.08 years. Most participants (55.2%) were single, followed by married individuals (42.1%). Educational attainment varied, with most participants holding an undergraduate degree (59.3%), while others had a secondary education (22.2%) or a postgraduate degree (18.5%). In terms of occupation, 54.3% were employed, 28.7% were students, 10.8% were unemployed, and 6.2% were retired. Income distribution revealed that 41.3% of participants earned less than 500 Bahraini Dinar (BHD), 23.5% earned between 501 and 1,000 BHD, 17.4% earned between 1,001 and 1,500 BHD, and 17.8% earned 1,500 BHD or more per month. BMI data indicated that 27.8% of participants had a normal BMI, 31.5% were overweight, and 40.7% had obesity (Table 1).

**Table 1:** Sociodemographic characteristics of the participants (*n* = 1,453).

Variable	*n* (%)
Sex	
Male	540 (37.2)
Female	913 (62.8)
Age (years)	
<25	502 (35.9)
26–35	441 (31.6)
36–45	256 (18.3)
>45	198 (14.2)
Marital status	
Single	802 (55.2)
Married	612 (42.1)
Divorced or widowed	39 (2.7)
Level of education	
Up to secondary	322 (22.2)
Undergraduate degree	861 (59.3)
Postgraduate degree	268 (18.5)
Occupation	
Student	417 (28.7)
Employed	788 (54.3)
Unemployed	156 (10.8)
Retired	90 (6.2)
Income	
<500 BD	600 (41.3)
501 to <1,000 BD	342 (23.5)
1,000 to <1,500 BD	253 (17.4)
≥1,500 BD	258 (17.8)
Body mass index (BMI)	
Normal	401 (27.8)
Overweight	455 (31.5)
Obesity	587 (40.7)

Table 2 shows a distribution of participants’ concerns about their weight. Among the participants who were asked about their motivation during their most recent attempt to lose weight, 21.8% reported being “slightly,” 21.9% “somewhat” concerned, 25.1% “moderately” concerned, and 19.9% “extremely” concerned, while only 11.3% reported being “not at all” concerned. Participants reported being “slightly” (29.8%), “somewhat” (12.3%), “moderately” (5.2%), and “extremely” (3.1%) concerned about their health because of being overweight; however, approximately half (49.6%) reported being “not at all” concerned. Among participants asked how important losing weight was as a life goal, 29.5% were “slightly” concerned, 18.4% “somewhat” concerned, 10.5% “moderately” concerned, and 8.0% “extremely” concerned, while approximately a third (33.5%) reported being “not at all” concerned.

**Table 2:** Perception of the participants regarding weight (*n* = 1,453).

	Not at all concerned *n* (%)	Slightly concerned *n* (%)	Somewhat concerned *n* (%)	Moderately concerned *n* (%)	Extremely concerned *n* (%)
What do you think is the motivation for your recent attempt to lose weight?	164 (11.3)	317 (21.8)	318 (21.9)	365 (25.1)	289 (19.9)
How concerned are you about your health because of being overweight?	720 (49.6)	433 (29.8)	179 (12.3)	76 (5.2)	45 (3.1)
Specify how important is losing weight as a goal in your life?	487 (33.5)	429 (29.5)	268 (18.4)	153 (10.5)	116 (8.1)

The majority strongly agreed that eating unhealthy food was a major cause of weight issues (44.2% “to a great extent”), followed by the perception that healthy food is expensive (38.4%). Approximately 30% reported difficulty in cooking healthy meals at home. Preferences for fast food and frequent restaurant ordering were less strongly endorsed, with only 20.4 and 24.2% respectively, agreeing “to a great extent.” Difficulty controlling overeating during special events was acknowledged by 25.8% to a great extent, while responses across other categories were more evenly distributed, indicating mixed attitudes toward behavioral contributors (Table 3).

**Table 3:** Participants’ agreement about behavioral factors, exercise-related factors, and dietary factors causing weight gain (*n* = 1,453).

	To a great extent	Somewhat	Neutral	Very little	Not at all
Eating unhealthy food is the reason for my weight gain	642 (44.2)	501 (34.5)	176 (12.1)	97 (6.7)	37 (2.5)
Eating healthy food is expensive for me	558 (38.4)	491 (33.8)	195 (13.4)	94 (6.5)	115 (7.9)
It is difficult for me to cook home-based healthy food for myself	435 (29.9)	422 (29)	205 (14.1)	159 (10.9)	232 (16)
I prefer to eat fast food	297 (20.4)	359 (24.7)	271 (18.7)	313 (21.5)	213 (14.7)
I prefer to order food from a restaurant on most days of the week	351 (24.2)	351 (24.2)	182 (12.5)	357 (24.6)	212 (14.6)
It is difficult for me to control overeating, especially during special events	375 (25.8)	413 (28.4)	229 (15.8)	231 (15.9)	205 (14.1)
Eating balanced food is the main strategy to lose weight	473 (36.26)	524 (36.1)	295 (20.3)	91 (6.3)	70 (4.8)
Exercising is the main strategy to lose weight	557 (38.3)	543 (37.4)	224 (15.4)	83 (5.7)	46 (3.2)
A lack of exercise is the reason for my weight gain	720 (49.6)	433 (29.8)	179 (12.3)	76 (5.2)	45 (3.1)
Performing exercise is expensive for me	127 (8.7)	375 (25.8)	340 (23.4)	204 (14)	407 (28)

**Figure 1:**
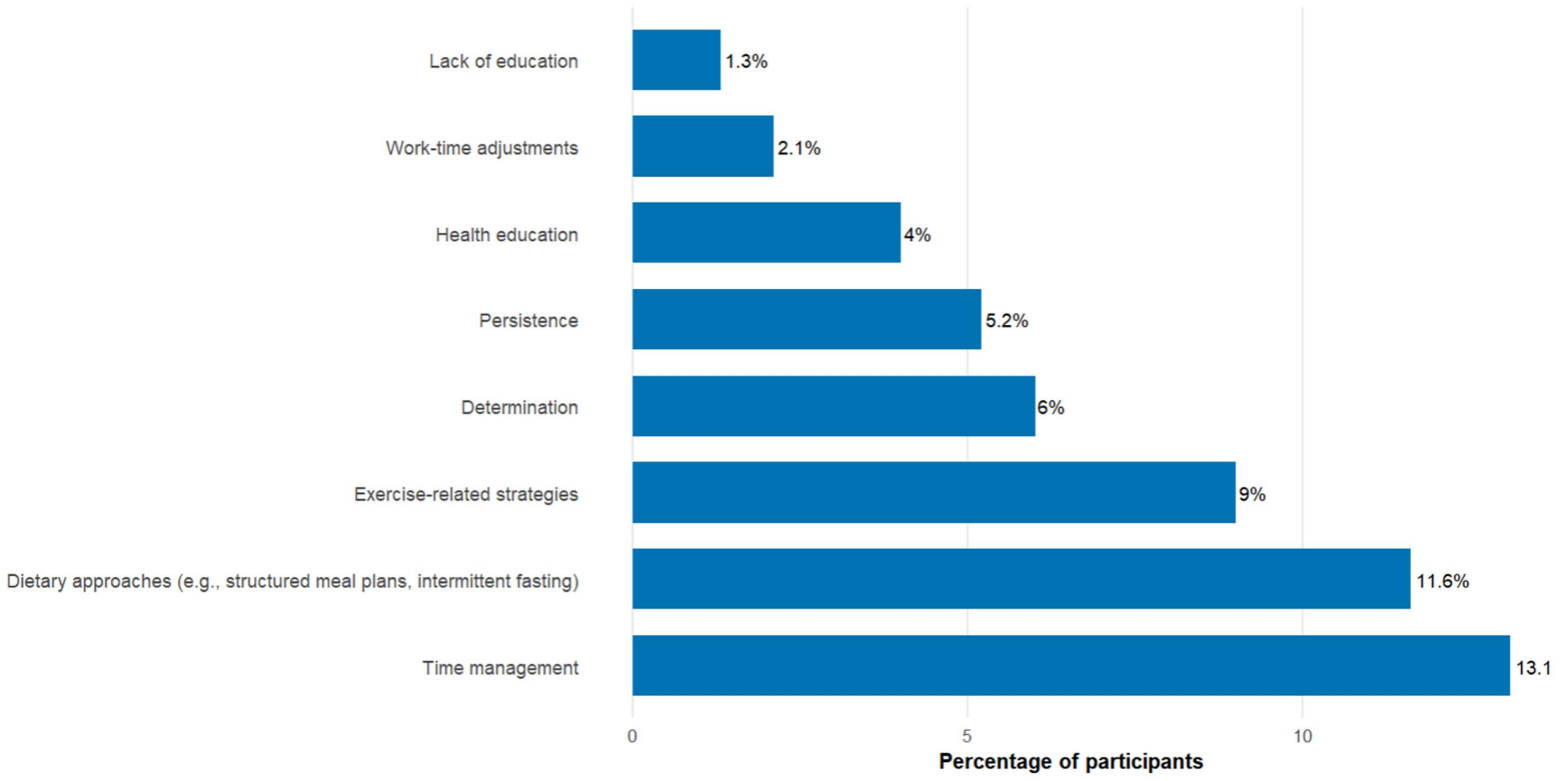
Participants’ perception of the strategies to overcome the barriers to weight loss (*n* = 1,453).

The bar chart displays the frequency (percentage) of respondents who identified each strategy as helpful in addressing weight-loss challenges. The most frequently reported strategies included time management (13.1%), dietary approaches such as structured meal plans or intermittent fasting (11.6%), exercise-related strategies (9.0%), determination (6.0%), and persistence (5.2%). Fewer participants cited health education (4.0%), work-time adjustments (2.1%), and lack of education (1.3%) as strategies (see Figure 1).

Table 4 presents logistic regression results for factors associated with overweight/obesity. The multivariable logistic regression model demonstrated a satisfactory overall fit to the data. The Hosmer–Lemeshow goodness-of-fit test yielded a non-significant result (*χ*^2^ = 7.82, *p* = 0.45), indicating that the model’s predicted probabilities were consistent with the observed outcomes and that there was no evidence of poor fit. The Nagelkerke *R*^2^ value of 0.312 suggests that approximately 31.2% of the variance in obesity status among participants was explained by the included covariates, which is considered acceptable for behavioral and health-related models. In addition, the likelihood ratio test (*χ*^2^ = 226.5, *p* < 0.001) confirmed that the model with the selected predictors provided a significantly better fit than the null model containing no predictors. Collectively, these indicators confirm that the final model demonstrated good explanatory power and adequately represented the relationships between sociodemographic, behavioral, and perceptual factors as well as obesity status (Table 4).

**Table 4:** Logistic regression of factors associated with overweight and obesity among Bahraini adults (*n* = 1,453).^a^

Characteristics	Crude odds ratio		Adjusted odds ratio	
	OR (95% C. I)	*p*-value	OR (95% C. I)	*p*-value
Sex				
Male	Ref.		Ref.	
Female	0.45 (0.35–0.59)	<0.001	0.50 (0.37–0.67)	<0.001
Age (years)				
<25	Ref.		Ref.	
26–35	2.26 (1.71–2.99)	<0.001	1.95 (1.31–2.91)	<0.01
36–45	4.77 (3.21–7.07)	<0.001	3.37 (1.91–5.95)	<0.001
>45	6.20 (3.84–9.99)	<0.001	3.59 (1.75–7.37)	<0.001
Marital status				
Single	Ref.		Ref.	
Married	2.88 (2.23–3.73)	<0.001	1.79 (1.30–2.46)	<0.001
Divorced or widowed	1.54 (0.73–3.24)	0.247	0.89 (0.39–2.01)	0.778
Level of education				
Secondary and below	Ref.		Ref.	
Undergraduate degree	1.02 (0.77–1.36)	0.848	0.79 (0.56–1.09)	0.160
Postgraduate degree	1.52 (1.04–2.22)	0.030	0.70 (0.43–1.14)	0.158
Occupation				
Student	Ref.		Ref.	
Employed	2.39 (1.85–3.08)	<0.001	1.49 (1.05–2.12)	0.022
Unemployed	2.63 (1.71–4.03)	<0.001	2.36 (1.48–3.76)	<0.001
Retired	12.42 (4.93–31.26)	<0.001	7.67 (2.86–20.53)	<0.001
Income				
<500 BD	Ref.		Ref.	
501 to <1,000 BD	1.55 (1.15–2.08)	<0.001	1.018 (0.709–1.46)	0.924
100 to <1,500 BD	2.09 (1.48–2.96)	<0.001	1.105 (0.721–1.69)	0.648
≥1,500 BD	2.80 (1.94–4.04)	<0.001	1.442 (0.903–2.30)	0.125
Primarily did dieting to lose weight				
No	Ref.		Ref.	
Yes	2.09 (1.55–2.80)	<0.001	2.534 (1.812–3.54)	<0.001
Primarily exercised to lose weight				
No	Ref.		Ref.	
Yes	1.31 (0.98–1.75)	0.061	1.478 (1.067–2.04)	0.019
Primarily used medications to lose weight				
No	Ref.		Ref.	
Yes	5.73 (3.28–10.00)	<0.001	5.233 (2.927–9.35)	<0.001
Underwent surgery to lose weight				
No	Ref.		Ref.	
Yes	2.02 (1.12–3.62)	0.018	1.905 (0.957–3.79)	0.066
Eating unhealthy food is the reason for my weight gain				
To a great extent	Ref.		Ref.	
Somewhat	1.07 (0.50–2.27)	0.856	1.116 (0.831–1.50)	0.465
Neutral	1.08 (0.51–2.31)	0.831	0.761 (0.510–1.13)	0.182
Very little	0.67 (0.30–1.49)	0.336	0.910 (0.541–1.53)	0.722
Not at all	0.88 (0.38–2.07)	0.785	1.099 (0.466–2.59)	0.829
A lack of exercise is the reason for my weight gain				
To a great extent	Ref.		Ref.	
Somewhat	0.78 (0.60–1.01)	0.066	0.80 (0.600–1.07)	0.137
Neutral	0.48 (0.33–0.71)	<0.001	0.59 (0.391–0.90)	0.015
Very little	0.76 (0.43–1.34)	0.359	0.80 (0.435–1.50)	0.499
Not at all	0.61 (0.33–1.13)	0.120	0.77 (0.393–1.52)	0.462

a Adjusted odds ratios were obtained from multivariable logistic regression models controlling for age, sex, marital status, education level, occupation, and income. Reference categories are indicated as ‘Ref.’.

**Figure 2:**
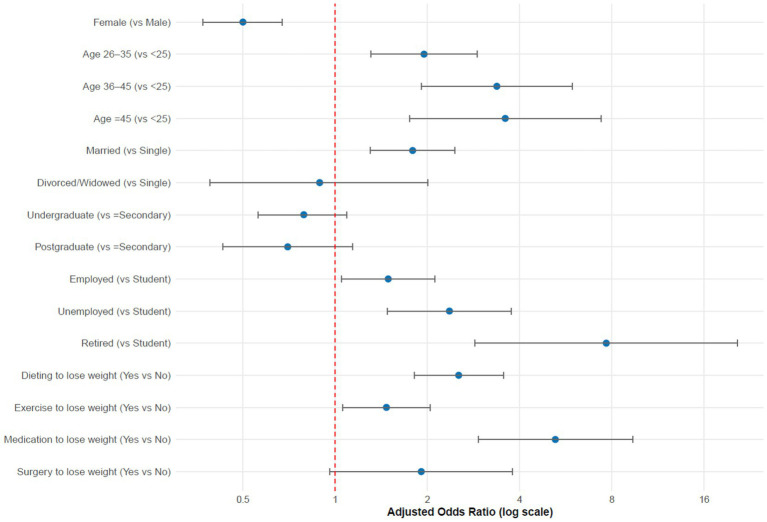
Adjusted odds ratio for factors associated with obesity (*n* = 1,453).

Although the crude prevalence of overweight and obesity was higher among women than men, adjusted analysis showed that women had significantly lower odds of obesity after controlling for age, marital status, occupation, income, and behavioral factors. This indicates that the higher raw prevalence among women was influenced by confounding demographic differences in the sample. Increasing age was associated with higher odds, notably among those aged 36–45 years (AOR = 3.37, 95% CI: 1.91–5.95) and >45 years (AOR = 3.59, 95% CI: 1.75–7.37). Married (AOR = 1.79, 95% CI: 1.30–2.46), unemployed (AOR = 2.36, 95% CI: 1.48–3.76), and retired individuals (AOR = 7.67, 95% CI: 2.86–20.53) had elevated odds. Those who dieted (AOR = 2.53, 95% CI: 1.81–3.54), exercised (AOR = 1.48, 95% CI: 1.07–2.04), or used medications (AOR = 5.23, 95% CI: 2.93–9.35) to lose weight were more likely to be obese. Neutral views on exercise-related weight gain showed reduced odds (AOR = 0.59, 95% CI: 0.39–0.90) (Table 4; Figure 2).

## Discussion

This study, which included Bahraini adults who had attempted weight loss within the past year, examined perceived barriers to achieving and maintaining an optimum weight. The cost of eating healthy food emerged as the most frequently reported barrier (38.4%). Regularly ordering food from restaurants was also common, with 24.2% largely agreeing and 24.2% somewhat agreeing that this behavior hindered weight loss. Furthermore, participants’ perceived time management (13.1%) was considered the most effective strategy to overcome barriers to weight loss. These behaviors were associated with obesity status and may reflect responses to living with obesity rather than causal factors, given the cross-sectional design.

Although the crude prevalence of overweight and obesity was higher among women (81.4%) than men (66.7%), the adjusted analysis indicated that female participants had significantly lower odds of obesity (AOR: 0.50, 95% CI: 0.37–0.67). This apparent discrepancy reflects the influence of confounding factors. Women in the sample were more likely to be younger and students—groups generally associated with a lower risk of obesity. Apparently, men were more represented in older and employed categories, which are linked to higher obesity risk. After adjusting for these sociodemographic factors, female sex remained a significant independent predictor, showing a protective effect against obesity. This result is an overestimate of the findings from other studies in the region. For example, a survey from Saudi Arabia showed an overweight and obesity rate of 64.1 in men and 53.1% in women (10). This difference is because we selected participants who were overweight or obese recently and had been trying to lose weight. Additionally, the finding that married (83.4%) and divorced/widowed (73.0%) participants had higher overweight and obesity rates compared to single individuals (63.6%), and that married individuals having a higher likelihood of obesity compared to singles (AOR: 1.79, 95% CI: 1.30–2.46) is consistent with existing literature that suggests that being married is associated with obesity as marital status may be associated with lifestyle and health behaviors, contributing to weight gain (10).

Educational attainment was another significant factor. A lower proportion of participants with secondary education or below (70.4%) were classified as obese or overweight compared to those with undergraduate (71.0%) and postgraduate degrees (78.4%) (*p* < 0.05). However, educational attainment did not show a statistically significant association with obesity in the multivariate model, which contrasts with the consensus in the literature that higher education is associated with lower obesity rates. However, in the univariate analysis, individuals with higher education had a slightly higher prevalence of obesity. These differences are negligible, as most of the local population has at least an undergraduate degree. Nevertheless, this finding suggests that other factors, such as socioeconomic status and access to health resources, may play a more critical role in this population. This is also possible because participants with higher education were older, with a longer duration to gain weight. This study and other studies from the region show that obesity is more prevalent in older people (11).

In this study, an unbalanced or unhealthy diet (29.2%) was identified as a contributing factor to weight gain. Additionally, participants who used dieting as a strategy to lose weight showed significantly higher odds of obesity (AOR: 2.53, 95% CI: 1.81–3.54). Although this could be due to reverse causality, where people with obesity are more likely to diet, studies have shown that while dieting is associated with short-term weight loss, many individuals struggle to maintain this weight over time, often regaining the weight they lost and sometimes even more. This cycle of weight loss and regain is associated with an overall increase in body weight and fat mass (12). Research indicates that repeated cycles of weight loss and gain can have adverse metabolic effects, making it harder to lose weight during subsequent attempts (13). Second, individuals who are already obese may be more likely to diet to lose weight, which could explain the higher incidence of obesity among those who report following a diet (14).

Dietary factors emerged as key barriers to achieving or maintaining weight in this study. The cost of eating healthy food emerged was the most important barrier, with 38.4% of participants agreeing to a great extent and 33.8% somewhat agreeing. This finding is consistent with previous studies highlighting the economic challenges associated with maintaining a healthy diet, where lower income has been associated to poorer weight loss outcomes (15). Additionally, the difficulty of preparing home-based healthy meals was reported as a barrier by 29.9% to a great extent and 29.0% somewhat agreeing, which is also consistent with studies showing that the time and effort can be constraints for preparing healthy meals (16). In this study, a small minority of the participants believed that junk food consumption (14.0%), contributed to weight gain, and preference for fast food was identified a significant barrier by 20.4% of participants to a great extent and 24.7% somewhat, consistent with studies that link fast food consumption with increased caloric intake and weight gain (14). A study showed that approximately 66.3% of Bahraini college students regularly consume fast food (17). The young people especially indulge in a diet rich in sugar, and processed food (17, 18). Regularly ordering food from restaurants was also common, with 24.2% agreeing to a great extent and 24.2% somewhat, further strengthening the evidence that a diet predominantly comprising fast food is associated with obesity (19).

A lack of physical activity (29.5%) and an unbalanced or unhealthy diet (29.2%) were identified as the main contributing factors in weight loss in this study. The identification of lack of exercise as a primary factor by 49.6% to a great extent and 29.8% somewhat, emphasizes the commonly held belief that weight gain is mainly because of a lack of physical activity (20). These findings align with previous research, which consistently shows that physical inactivity and poor dietary habits are the significant contributors to weight gain (13, 21). However, in this multivariate model, this association was not statistically significant; those who believed lack of exercise was a major cause of weight gain had a significantly lower likelihood of obesity (AOR: 0.802, 95% CI: 0.600–1.072), emphasizing the importance of physical activity in weight management. Previous literature has also identified a lack of physical activity as a perceived barrier to weight loss (12, 22, 23). Gym memberships, workouts, group training, and exercise support groups were noted by 9.0% of participants as other barriers, emphasizing the value of physical activity and social support in weight loss efforts. Determination (6.0%) and persistence and continuity (5.2%) were also significant, aligning with studies that highlight the psychological resilience required for sustained weight loss (22).

In this study, a small proportion of the participants believed that stress, emotional eating, and mental illness (8.6%) contribute to weight gain. These factors are frequently cited in the literature as contributing to obesity, with stress and emotional eating being particularly significant in modern, high-stress environments (24). Moreover, controlling overeating during special events was challenging for 25.8% of participants to a great extent and 28.4% somewhat, highlighting the social and psychological dimensions of eating behavior (25). Food-related unhealthy behavior is common in the region to the extent more than half of the families buy ready-made food for an early morning meal in the month of Ramadan, as study from the region reported (26). Also, during guest hospitality, it is a tradition to serve more food than the guest is expected to eat, and the refusal to eat or drink is a sign of unacceptance of the host (27). Additionally, regional culture is increasingly adopting a modern and westernized lifestyle (1). Lastly, the recognition of long working hours (5.6%) further identifies the association of work habits on weight gain, also observed in other studies (28).

The analysis revealed that participants who reported taking medication for weight loss had a significantly increased likelihood of obesity (AOR: 5.233, 95% CI: 2.927–9.356), suggesting that individuals with obesity were more likely to be using weight loss medications compared to normal weight. This can be explained by several factors and is supported by existing literature. First, this finding shows the challenges associated with long-term weight management, as medications for weight loss are often prescribed to individuals who have struggled with obesity for a long period of time and have not succeeded with nonmedical weight loss methods. These individuals may also have more severe obesity and related health issues, which could explain the higher likelihood of obesity among those taking weight-loss medications (29). Second, the use of weight-loss medications might indicate a more severe underlying metabolic condition that makes weight loss particularly challenging for these individuals. While weight-loss medications can be effective in the short term, they often need to be combined with lifestyle changes such as diet and exercise to sustain long-term weight loss. Individuals who do not use weight-loss medications must rely on sustainable and effective long-term weight management lifestyle strategies, including balanced diets and regular physical activity (30).

Occupational status further highlighted disparities as one of the causes for obesity. Unemployed participants (78.1%) had a substantially higher obesity rate compared to employed individuals (76.4%) and students (57.5%). Employment status was a significant factor, with unemployed participants having a higher likelihood of obesity compared to students. This is because unemployment is associated with reduced financial resources, negatively impacting dietary choices and physical activity levels (31). This suggests that unemployment may be associated with increased stress, which can negatively impact dietary choices and physical activity levels (31). Participants with lower incomes (<500 BD) had lower obesity rates (64.0%) than those compared with higher income brackets; however, the relationship was not linear. This trend is not consistent with the socioeconomic gradient in health, where lower income is often linked to higher obesity rates because higher income individuals may also have more resources to invest in healthier food options and fitness activities, thereby reducing their risk of obesity (32, 33).

### Strengths and limitations

This study is among the first in the region to examine weight-loss experiences among individuals who attempted weight reduction in the past year. The questionnaire was developed through a comprehensive literature review, validated by 10 experts, and pretested for internal consistency. Administering both English and Arabic versions enhanced inclusivity, while social media recruitment enabled broad participation. Limitations of the study include reliance on self-reported data, the cross-sectional design limiting causal inference, potential sampling bias from online recruitment, and a short data collection period that may not capture seasonal variations. This study focused only on adults who had attempted weight loss in the past year, thereby introducing selection bias and limiting generalizability. The findings should therefore be interpreted as reflecting the behaviors and perceptions of individuals actively engaged in weight management. The online cross-sectional design using social media recruitment may have likely resulted in a non-representative sample, skewed toward younger and more educated participants, and limits the generalizability of the findings.

## Conclusion

This study identifies a high prevalence of overweight and obesity in Bahrain, particularly among men, driven by unhealthy dietary patterns, physical inactivity, and socioeconomic disparities. Despite widespread concern about weight, awareness of associated health risks remains limited. To address these challenges, public health strategies should prioritize affordable access to nutritious foods, culturally tailored education campaigns, and inclusive physical activity options. Workplace-based wellness programs and community-driven initiatives may further support sustainable behavior change. Future research should explore long-term weight management approaches, cultural perceptions, and prevalent misconceptions to inform context-sensitive interventions and guide policy development aimed at reducing the burden of obesity and related NCDs.

## Data Availability

The raw data supporting the conclusions of this article will be made available by the authors, without undue reservation.
